# Ammonium Ferric Citrate induced Ferroptosis in Non-Small-Cell Lung Carcinoma through the inhibition of GPX4-GSS/GSR-GGT axis activity

**DOI:** 10.7150/ijms.54860

**Published:** 2021-03-03

**Authors:** Wei Wu, Zixiang Geng, Haoran Bai, Te Liu, Bimeng Zhang

**Affiliations:** 1 Department of Anesthesiology, Shanghai Pulmonary Hospital, Tongji University School of Medicine, Shanghai 200433, China.; 2 Department of Acupuncture, Shanghai General Hospital, Shanghai Jiao Tong University School of Medicine, Shanghai 200086, China.; 3 Department of Oncology, Longhua Hospital, Shanghai University of Traditional Chinese Medicine, Shanghai 200031, China.; 4 Shanghai Geriatric Institute of Chinese Medicine, Shanghai University of Traditional Chinese Medicine, Shanghai 200031, China.

**Keywords:** ammonium ferric citrate, non-small-cell lung carcinoma, autophagy, ferroptosis

## Abstract

The morbidity and mortality rates associated with non-small-cell lung carcinoma (NSCLC) are increasing every year, placing new demands on existing therapies and drugs. Ammonium ferric citrate (AFC) is often used as a food additive for iron supplementation; however, to our knowledge, no studies have investigated whether AFC can induce ferroptosis in NSCLC. In this study, we demonstrated that specific concentrations of AFC effectively inhibit the proliferation and invasion of lung cancer cell lines* in vitro* using a cell proliferation inhibition test, a transwell assay, and flow cytometry analysis of cell cycle and apoptosis. In addition, AFC significantly induced oxidative stress injury in lung cancer cell lines. A quantitative polymerase chain reaction assay showed that AFC markedly reduced the expression levels of cell growth factors, negative regulators of ferroptosis, and autophagy regulators. Lastly, a protein-protein interaction analysis revealed that glutathione peroxidase 4 (GPX4) exerted its biological role through the regulation of the GSS/GSR complex and downstream GGT family proteins. When the expression of GPX4 changes, its biological activities, such as the glutathione metabolic process, cellular biosynthetic process, cellular response to chemical stimulus, and antioxidant activity, change accordingly, thereby affecting the survival quality and physiological and biochemical activities of cells. Overall, this study verifies that AFC has the biological activity of activating oxidative stress injury in NSCLC cell lines, leading to a decrease in their autophagy and inducing ferroptosis. We also confirmed that the GPX4-GSS/GSR-GGT axis is a crucial target of AFC-induced ferroptosis.

## Introduction

Lung cancer is one of the most common malignant tumours worldwide and has become the primary cause of death from malignant tumours in the urban population in China 1-4. Non-small-cell lung carcinoma (NSCLC) is a group of lung malignancies originating from the bronchial mucosa, bronchial glands, and alveolar epithelium, and includes squamous cell carcinoma, adenocarcinoma, and large cell carcinoma. NSCLC accounts for 80% of all lung cancers, and approximately 75% of patients are found to be in an intermediate to advanced stage, with a low five-year survival rate 1-4. Usually, surgical therapy combined with radiotherapy and chemotherapy is the preferred and primary therapy for lung cancer 1-4. However, chemotherapy generally does not cure NSCLC and only prolongs survival and improves the quality of life. Additionally, NSCLC is not sensitive to radiotherapy 1-4. Therefore, it is essential to seek more effective treatments for NSCLC.

Ammonium ferric citrate (AFC), also known as 2-hydroxy-1,2,3-propanetricarboxylic acid ferric ammonium salt, is a double salt of ferric citrate and ammonium citrate 5-8. AFC is usually added to dairy products, wheat flour for bread-making, and biscuits and milk powder as a nutritional supplement (iron fortifier) 5-8. It is also used as a hematinic to treat iron deficiency anaemia. Moreover, AFC can be used for microbial cultures, such as to add to JFM media to culture ferromanganese bacteria 5-8. When exposed to sunlight, the Fe^3+^ in citrate is reduced to Fe^2+^, which produces Turnbull's blue in the presence of water; thus, AFC can also be used for photo printing 5-8. In *Physalisalkekengi* L.var.franchetii (Mast.) Makino, the AFC content is very high, thus, the fruit is beneficial for the prevention of a plastic anaemia 9. AFC is normally used as a food additive, but its anti-cancer effects have not yet been reported.

Autophagy is a process in which a cell engulfs its own cytoplasmic proteins or organelles and envelops them into vesicles that fuse with lysosomes to form autolysosomes, and degrades their contents, thereby realizing the metabolic needs of the cell and the renewal of certain organelles 10-14. Autophagy is involved in both physiological and pathological processes of the body, and it is particularly important for the proliferation and invasion of tumour cells. The inhibition of autophagy significantly diminishes the self-renewal and invasive functions of tumour cells 15-20. During normal cellular activities, such as metamorphosis, senescence, and differentiation in animals, autophagy has been found to be responsible for the degradation of normal proteins to reconstitute cells 10, 16, 18-20. However, under some pathological and pressure conditions, cells may selectively isolate some organelles, including mitochondria and peroxisomes, through autophagy 14, 15, 20. In the starvation response, the lack of amino acids in different organs, such as the liver, induces autophagy, which breaks down macromolecules to produce intermediate metabolites necessary for catabolic and anabolic processes. Therefore, autophagy is essential for body renewal, cell division, and resistance to harsh environments 10, 11, 19, 20. For tumour cells, the induction of autophagy inhibition is undoubtedly an effective treatment.

Ferroptosis is an iron-dependent, novel mode of programmed cell death that is distinct from apoptosis, necrosis, and autophagy 20-25. The main mechanism of ferroptosis is that divalent iron or ester oxygenase catalyses unsaturated fatty acids highly expressed on the cell membrane to cause liposome peroxidation, thus, inducing cell death; it is also shown in a decrease in GPX4 expression in the antioxidant system (lipid peroxides) 20-25. GPX4 is the sole glutathione peroxidase (GPX) used in liposome peroxides 25. During lipid peroxidation, GPX4 converts peroxy bonds to hydroxyl groups; therefore, the activity of peroxides is lost. It has been suggested that when iron is assimilated into cells and exists in the divalent form, liposome peroxidation is initiated in an iron overload state. Peroxidised liposomes significantly digest the activity of GPX4 *in vivo* and induce ferroptosis 25. When cells undergo ferroptosis, they have smaller mitochondria, increased membrane density, and reduced cristae, accompanied by increased lipid peroxidation and elevated reactive oxygen species (ROS) in the cytoplasm. It has previously been revealed that ferroptosis dramatically promotes the death of cancer cells and inhibits cancer cell proliferation, division, and invasion; however, whether AFC induces ferroptosis has not yet been fully elucidated.

Based on the above results, we investigated whether AFC inhibits the activity of NSCLC by regulating autophagy and ferroptosis.

## Materials and Methods

### Cell cultured

The human lung adenocarcinoma cell line A549 HCC827, NCI-H1299 and NCI-H661 were grown in DMEM (Hyclone, Logan, USA) supplemented with 10% fetal bovine serum (FBS) (PAA Lab. Inc., Queensland, Australian), penicillin (100 U/ml), streptomycin (100 U/ml), and 2 mM L-glutamine (all from Hyclone). A549, HCC827, NCI-H1299 and NCI-H661 were maintained at 37 °C in a humidified atmosphere of air containing 5% CO_2_.

### MTT assay

The MTT assay was performed as previously described 26, 27. Briefly, 2000 cells/ml were seeded in a 96-well cell culture plate. After 24 h, 10 μl of 3-(4,5-dimethylthiazol-2-yl)-2,5 diphenyl tetrazolium bromide solution (Sigma-Aldrich, St. Louis, USA) was added to the cells in each group, and plates were incubated for 3 h at 37 °C. The conditioned medium was discarded, and 150 μl of dimethyl sulfoxide (Sigma-Aldrich) was added to each well and thoroughly mixed by shaking for 15 s. The culture plates were placed in a microplate reader, and absorbance at 450 nm was recorded. The formula for the calculating the inhibition of cell proliferation (%) was as follows: (1-OD value of the experimental group cells-control/OD value of the control group cells-blank)×100%.

### RNA extraction, RNA extraction with reverse transcription reaction, and quantitative polymerase chain reaction (qPCR)

According to the instructions of RNAprep pure Tissue Kit (TIANGEN Biotech (Beijing) China) Co., Ltd, Beijing, China), about 20 mg of human tissue specimen was added 800 μL of lysis solution, ground, and homogenized. To the supernatant, 200 μL of chloroform was added, mixed by inverting, and centrifuged at 4 °C at 13400 ×* g* for 15 min. To the obtained supernatant, two volumes of absolute ethanol were added, mixed by inversion, and centrifuged at 4 °C at 13400 × *g* for 30 min. Ethanol precipitation and centrifugation of the supernatant was repeated. The RNA pellet was added with 500 μL of 75% ethanol and centrifuged at 13400 × *g* at 4 °C for 5 min. The supernatant was discarded, the excess liquid was removed, and the pellet was dissolved in 300 μL of DECP water. One microliter of the RNA solution was used to detect the ratio of OD260/OD280 (generally controlled between 1.8~2.0) to determine the purity and concentration of the RNA. RNA samples were treated with DNase I (Sigma-Aldrich), quantified, and reverse-transcribed into cDNA using the ReverTra Ace-α First Strand cDNA Synthesis Kit (Toyobo). Quantitative real-time PCR (qPCR) was conducted using a RealPlex4 real-time PCR detection system (Eppendorf, Germany) with SYBR Green Realtime PCR Master Mix (Toyobo). On a quantitative real-time PCR instrument, the following reactions were performed: 95 °C for 15 min; 94 °C for 20 sec; 60 °C for 34 sec, and the fluorescence value was read. The above reactions were performed for 40 cycles.

### Western blot

With reference to previous studies 14, 26, 27, the total proteins from each group of cells were used for 12% sodium dodecyl sulphate-polyacrylamide denaturing gel electrophoresis, then transferred to polyvinylidene difluoride membranes (Millipore). After sealing and washing the membrane, primary antibodies were incubated at 37 °C and allowed to react for 45min. After fully washing the membrane, secondary antibodies were incubated at 37 °C and allowed to react for 45 min. The membranes were washed four times with TBST at room temperature for 14 min each time. They were then exposed to development (Sigma-Aldrich Chemical) using ECL-enhanced chemiluminescence (ECL kit, Pierce Biotechnology).

### Transwell migration assay

First, 200 μl of serum-free cell culture media containing 2,000 cells/ml was inoculated into the upper chamber of an 8 μm/well Transwell. Then, 600 μl of complete media containing 10% foetal bovine serum was inoculated into the lower chamber of the Transwell. Cells were cultured at 37 °C and 5% CO_2_ for 48 h. Cells adhering to the membrane surface were fixed with 4% paraformaldehyde at room temperature for 30 min and stained with 4, 6-diamidino-2-phenylindole (Sigma-Aldrich Chemical) for 10 min. Three non-overlapping fields of view were selected under a microscope to calculate the total number of cells.

### Propidium iodide (PI) staining and flow cytometry identification

With reference to previous studies 26, 27, 5 × 10^5^ cells/ml were collected and fixed in 1 ml of 70% ice-precooled ethanol for 48 h. The cells were centrifuged at 1,500 r/min, 4 °C for 5 min. Then, the cell precipitate was collected, and PI staining solution (Sigma Chemicals) was added to react at 4 °C without light for 30 min. The cell cycle distribution of each group was analysed using flow cytometry (BD FACSAria), and the data were analysed using CellQuest software.

### Annexin V-FITC/PI staining and flow cytometry detection

Following the manufacturer's instructions for the Annexin V-FITC Apoptosis Detection Kit (Beytime biotechnology, China), the adherent cells were digested into single cells using trypsin, phosphate-buffered saline (PBS) was used to wash the cells once, and the residual body fluid was removed by centrifugation. Then, 195 μl of Annexin V-FITC binding solution was added to gently resuspend the cells and 5 μl of Annexin V-FITC was added. This solution was then gently mixed. Finally, 10 μl of PI staining solution was added and gently mixed. This solution was then incubated at 20 °C without light for 30 min and detected using flow cytometry (Cytomics FC 500, BECKMAN).

### ATP assay

Following the manufacturer's instructions of the enhanced ATP detection kit (Beyotime, Shanghai, China), 1 × 10^6^ cells/ml were retrieved,200 μl of lysis buffer specimen was added, which was then fully blown and centrifuged at 4 °C, 12,000×*g* for 5 min, after which the supernatant was collected. Meanwhile, an ATP standard control was set up and adjusted to concentrations of 0.01, 0.03, 0.1, 0.3, 1, 3, and 10 μM, which were detected at the same time as the specimen. Fresh assay working solution was prepared subject to the requirements of the kit. Then the 100 μl ATP assay working solution was added to the assay and specimen wells and incubated at room temperature for 5 min. The specimen or the standard (20 μl) was then added to the assay wells, mixed quickly, and placed at room temperature for 5 s. RLU values were measured using a Luminometer.

### ROS assay

Following the instructions of the ROS detection kit (Beyotime, Shanghai, China), DCFH-DA was diluted with serum-free media by 1:1000 to obtain a final concentration of 10 μmol/L. The cells were digested with trypsin and centrifuged to collect the cellular precipitate. The diluted DCFH-DA was added to adjust the final cell concentration to 1 × 10^6^ ml and incubated at 37 °C for 20 min. The cells were washed with serum-free cell culture media three times. A flow cytometer was used and FL1 channel (488 nm excitation wavelength, 525 nm emission wavelength) was selected to detect and count the intracellular fluorescence, counting 10,000 cells per sample.

### SOD assay

Following the manufacturer's instructions for the SOD Activity Detection kit (Beyotime, Shanghai, China), 1 × 10^6^ cells/ml were retrieved, and 200 μl of lysis buffer specimen was added. The mixture was then fully blown and centrifuged at 4 °C, 12,000×*g* for 5 min and the supernatant was collected. The WST-8/enzyme working solution was obtained by thoroughly mixing 151 μl of SOD detection buffer, 8 μl of WST-8, and 1 μl of enzyme solution. A concentration gradient of the SOD standard control was also established, and the SOD standard was diluted to 100, 50, 20, 10, 5, 2, and 1 U/ml, which were assayed simultaneously. The supernatant of the 20 μl cell lysis buffer and 20 μl standard solution was retrieved and separately added to 160 μl of freshly-prepared WST-8/enzyme working solution and 20 μl of reaction starter solution, mixed thoroughly, and incubated at 37 °C for 30 min. The absorbance was measured at 450 nm.

### Statistical analysis

Each experiment was performed as least three times; data are presented as mean ± the standard error (SE) where applicable. Differences were evaluated using Student's t-tests. P values < 0.05 were considered statistically significant.

## Results

### AFC significantly inhibited the proliferation and invasion of NSCLC cell lines *in vitro*

**Figure 1:**
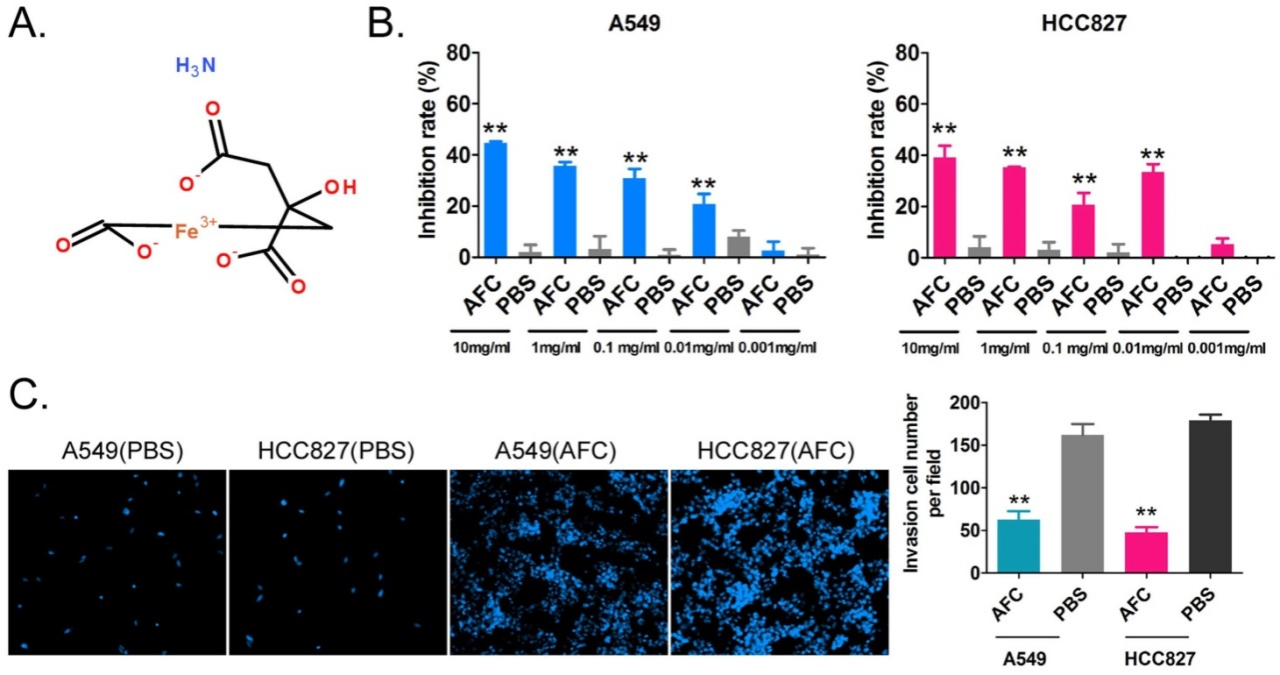
** AFC significantly inhibited the proliferation and invasion of the NSCLC cell line *in vitro*. (A)** The molecular structure of AFC. **(B)** The MTT assay results showing that AFC significantly inhibited the proliferation of the NSCLC cell line *in vitro*. **p < 0. 01 vs. PBS control group, t-test, n=3. **(C)** The transwell assay results showing that AFC significantly inhibited the migration of the NSCLC cell lines *in vitro*. **p < 0. 01 vs. PBS control group, t-test, n=3.

The NSCLC cell lines A549 and HCC827 were treated with different concentrations of AFC (10, 1, 0.1, 0.01, and 0.001 mg/ml) for 24 h, and an equal volume of PBS was used as a control. The results of the MTT assay suggested that except for 0.001mg/ml, the other four concentrations significantly inhibited the *in vitro* proliferation of the above-mentioned cell lines (Figure 1A, 1B). In order to further explore whether the inhibition of cell proliferation induced by AFC was caused by ferroptosis, the inhibitors of various cell death types (Z-DEVD-FMK was a specific caspase-3 inhibitor so as to inhibit cell apoptosis; Z-WEHD-FMK is a potent, cell-permeable and irreversible caspase-1/5 inhibitor so as to inhibit cell necrosis; Ferrostatin-1 (Fer-1), a potent and selective ferroptosis inhibitor so as to inhibit cell ferroptosis) were used in rescue experiments. Meanwhile, considering the effect of endogenous GPX4 expression, two lung cancer cell lines with GPX4 high-expression NCI-H1299 and NCI-H661 were used as positive control. The results of MTT assay showed there were no significant difference in the inhibition rates of cell proliferation of NSCLC cell lines between combination of Z-DEVD-FMK (12 μM) and AFC (1 mg/ml) treated, and AFC (1 mg/ml) alone treated (Figure S1). And, there were no significant difference in the inhibition rates of cell proliferation of NSCLC cell lines between combination of Z-WEHD-FMK (4 μM) and AFC (1 mg/ml) treated, and AFC (1 mg/ml) alone treated (Figure S1). However, the inhibition rates of cell proliferation of NSCLC cell lines treated with combination of Fer-1 (40 nM) and AFC (1 mg/ml) were significantly lower than them on control group (Figure S1). Additionally, the transwell assay showed that the number of migrating cells in A549 and HCC827 cell lines treated with AFC at 1mg/ml for 24 h was significantly lower than in the PBS control group (Figure 1C). The results indicated that AFC significantly inhibited the proliferation and invasion of NSCLC cell lines *in vitro*.

### AFC significantly inhibited cell cycle progression in NSCLC cell lines

**Figure 2:**
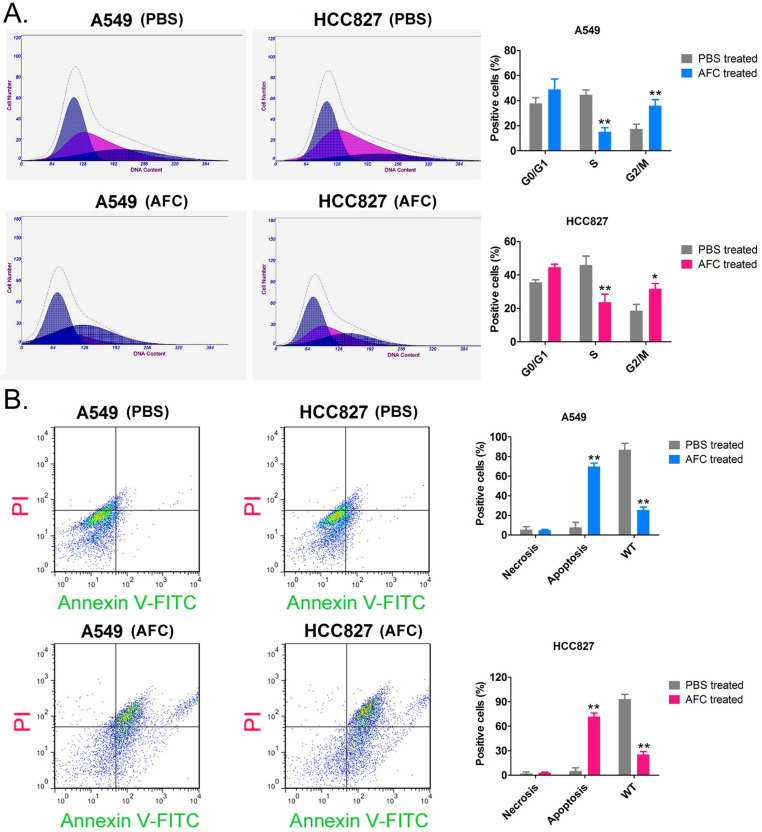
** AFC significantly inhibited cell cycle progression in NSCLC cell lines. (A)** PI staining/FCM assay results showing that AFC significantly inhibited cell cycle progression in the NSCLC cell lines. **p < 0.01 vs. PBS control group, *p < 0.05 vs. PBS control group, t-test, n=3. **(B)** Annexin V-FITC staining/FCM assay results showing that AFC significantly promoted apoptosis in NSCLC cell lines. **p < 0. 01 vs. PBS control group, t-test, n=3.

The results of the PI staining/FCM assay showed that after A549 and HCC827 cell lines were treated with 1 mg/ml AFC for 24 h, the number of cells in the S phase was significantly lower than in the control group, while the number of cells in the G2/M phase was significantly higher than in the control group (Figure 2A). This indicated that AFC blocked the cell cycle in the G2/M phase, thus, inhibiting cell division. In addition, Annexin V-FITC/PI staining and the FCM assay results showed that the proportion of normal cells appreciably decreased, while that of apoptotic cells perceivably increased in AFC-treated cells (Figure 2B). The results indicated that AFC significantly inhibited cell cycle progression in NSCLC cell lines and induced apoptosis.

### AFC caused elevated Fe^2+^ content in NSCLC cell lines and induced oxidative stress injury

**Figure 3:**
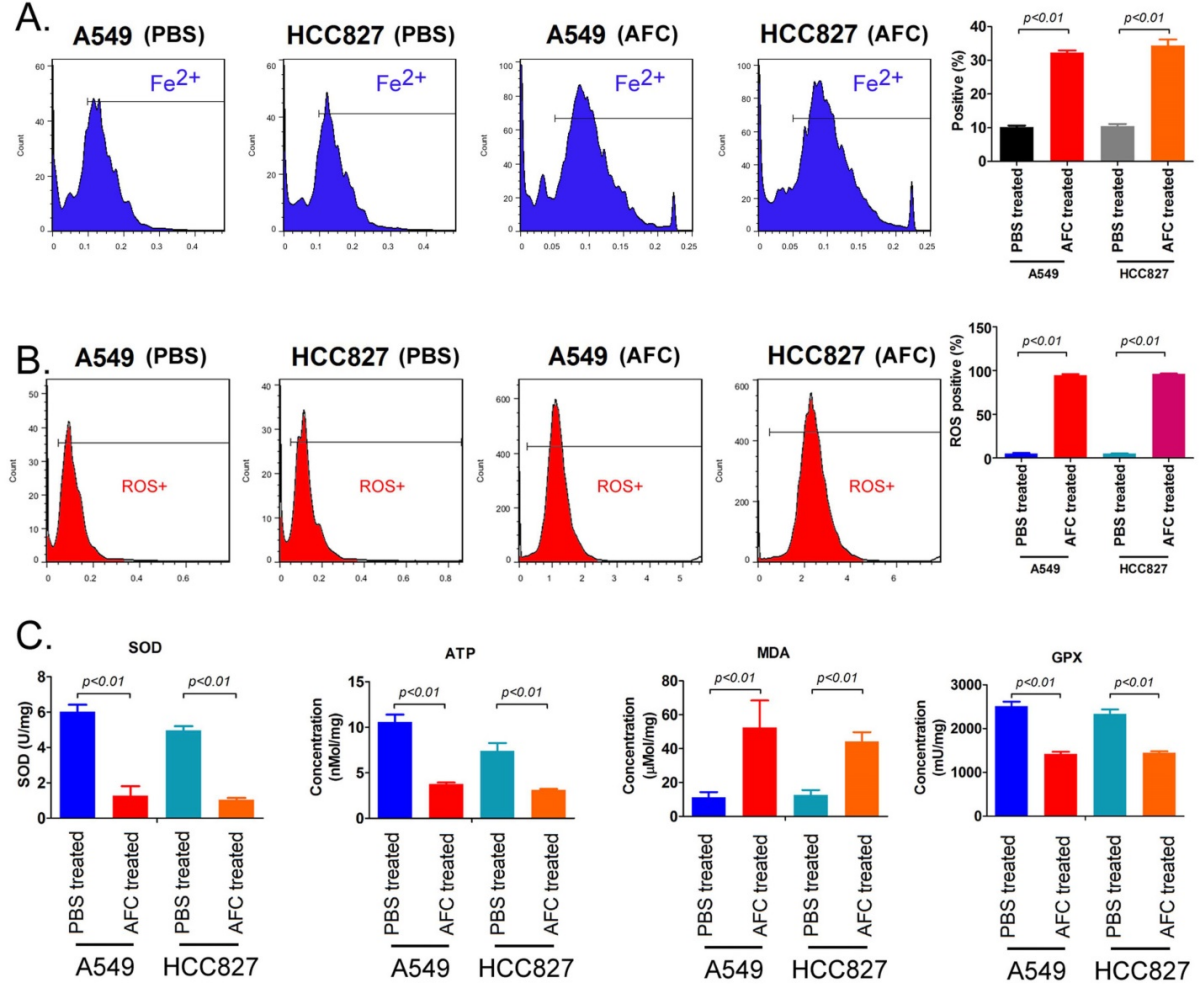
** AFC caused elevated Fe^2+^ content in NSCLC cell lines and induced oxidative stress injury. (A)** The FCM assay results showing that AFC significantly elevated the Fe^2+^ content in NSCLC cell lines. **(B)** The FCM assay results showing that AFC significantly increased the ROS levels in NSCLC cell lines. **(C)** The biochemical assay results showing that AFC significantly increased the MDA content in NSCLC cell lines but significantly decreased SOD, ATP, and GPX levels in the NSCLC cell lines.

FeRhonox-1 probe staining was used to identify the Fe^2+^ content in the cells of each group. The results of FeRhonox-1 probe staining and FCM assay showed that the percentage of positive cells on FeRhonox-1 probe staining in A549 and HCC827 cell lines treated with 1mg/ml AFC for 24 h was significantly higher than them in the control group (Figure 3A). Moreover, the results of FCM assay revealed that the the percentage of positive cells on ROS probe staining in A549 and HCC827 cell lines treated with 1mg/ml AFC for 24 h was appreciably higher than them in the control group (Figure 3B). Additionally, the results demonstrated that the MDA levels in the AFC-treated group were appreciably higher than those in the PBS control group (Figure 3C). However, the SOD, ATP, and GPX levels were significantly lower in the AFC-treated group than in the PBS control group (Figure 3D, 3E). In addition, the concentrations of lipid peroxidation (LPO) in NSCLC cell lines (A549, HCC827, and two cell lines NCI-H1299 and NCI-H661 with high expression of GPX4) treated with 1 mg/ml AFC were significantly higher than them in control group (Figure S1). However, the concentrations of LPO of NSCLC cell lines treated with AFC (1 mg/ml) and Fer-1 (40 nM) were significantly reduce, compared to them on control group (Figure S1). Meanwhile, the results indicated that GPX4 activities of NSCLC cell lines (A549, HCC827, and two cell lines NCI-H1299 and NCI-H661 with high expression of GPX4) treated with AFC (1 mg/ml) were significantly reduced, compared to them on control group (Figure S1). However, the GPX4 activities of NSCLC cell lines treated with AFC (1 mg/ml) and Fer-1 (40 nM) were significantly elevated, compared to them on control group (Figure S1). The results indicated that AFC caused elevated Fe^2+^ content in NSCLC cell lines and induced oxidative stress injury.

### AFC promoted differential gene expression profiles of proliferation, autophagy, and ferroptosis in NSCLC cell lines

**Figure 4:**
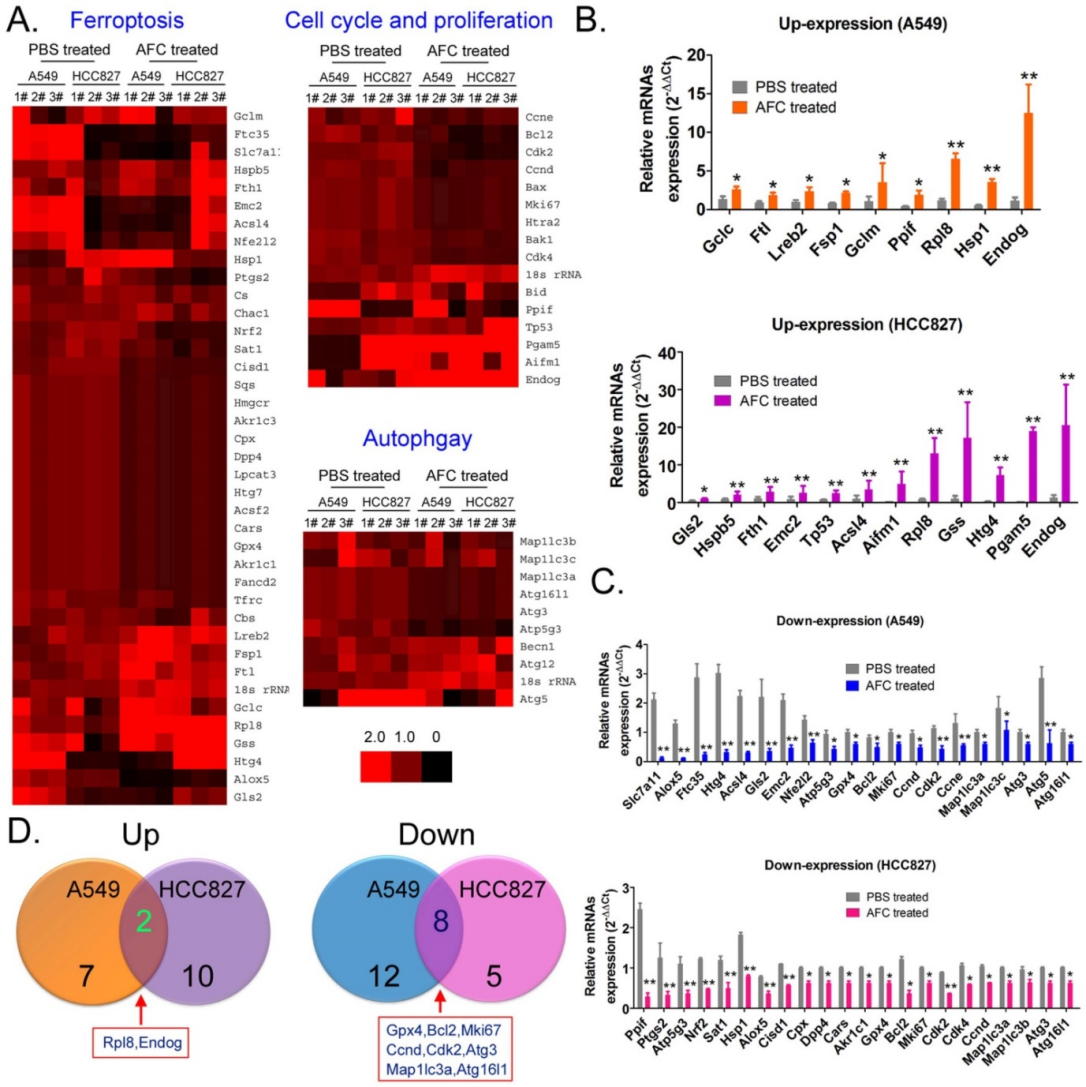
** AFC promoted differential gene expression profiles of proliferation, autophagy, and ferroptosis in NSCLC cell lines. (A)** Heat map of qPCR assay results. **(B)** The qPCR assay results showing that the mRNA expression levels of some cell proliferation inhibitors and ferroptosis promoting factors were significantly increased in the AFC cell group. **p < 0.01vs. PBS control group, *p < 0.05 vs. PBS control group, t-test, n=3. **(C)** The qPCR assay results showing that the mRNA expression levels of some cell cycle proliferation factors, autophagy promoting factors, and negative regulators of ferroptosis were significantly decreased in the AFC cell group. ** *p* < 0.01vs. PBS control group, *p < 0.05 vs. PBS control group, t-test, n=3. **(D)** A comprehensive analysis showing that the mRNA expression levels of two genes were significantly upregulated, and those of eight genes were significantly downregulated in AFC-treated NSCLC cell lines.

qPCR was used to analyse mRNA expression profiles (Figure 4A) involving ferroptosis, autophagy, and cell proliferation and death in A549 and HCC827 cell lines in the AFC-treated and PBS control groups at 24 h. The expression differences of 63 genes between the two groups were examined, with (AFC_gene_2^-ΔΔCt^/PBS_gene_2^-ΔΔCt^) ≥ 1.5 considered to have significantly increased expression levels and (AFC_gene_2^-ΔΔCt^/PBS_gene2^-ΔΔCt^) ≤ 0.5 significantly decreased expression levels (Table S1). The genes with significantly increased expression levels primarily included cell proliferation inhibitors, cell death proteins (A549: Endog; HCC827: Tp53, Aifm1, Pgam5, Endog), and some positive regulators of ferroptosis (Figure 4B). The genes with significantly decreased expression levels primarily involved cell cycle regulatory proteins, cell growth factors (A549: Bcl2, Mki67, Ccnd, Cdk2, Ccne; HCC827: Bcl2, Mki67, Cdk2, Cdk4, and Ccnd), autophagy-associated factors (A549: Map1lc3a, Map1lc3c, Atg3, Atg5, Atg16l1; HCC827: Map1lc3a, Map1lc3b, Atg3, and Atg16l1), and some negative regulators of ferroptosis (Figure 4C). A comprehensive analysis showed that only two genes, Endog and Rpl8, were upregulated in A549 and HCC827 cell lines treated with AFC. However, eight genes, namely, Gpx4, Bcl2, Mki67, Ccnd, Cdk2, Map1lc3a, Atg3, and Atg16l1, were downregulated in the two cell lines (Figure 4D). The results indicated that AFC significantly inhibited the expression of cell cycle regulators and autophagy initiation factors and promoted the expression of cell proliferation inhibitors and positive regulators of ferroptosis.

### AFC significantly affected the expression of the GPX4-GSS/GSR-GGT axis in NSCLC cell lines

**Figure 5:**
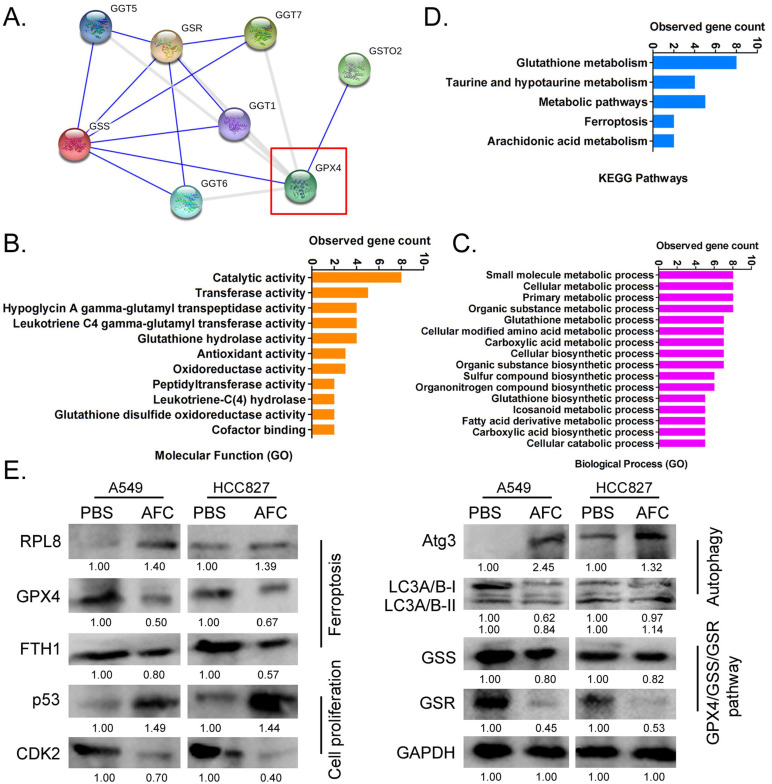
** AFC promoted differential gene expression profiles of proliferation, autophagy, and ferroptosis in NSCLC cell lines. (A)** A protein-protein interaction network prediction software result showing that GPX4 catalysed the expression of downstream GSS/GSR-GGT axis. **(B)** GO analysis results showing that the expression level of GPX4 significantly affected biological processes, such as small molecule metabolic processes, as well as molecular functions, such as catalytic activity. **(C)** KEGG pathway analysis results showing that GPX4 was highly correlated with the regulation of glutathione metabolism and ferroptosis signalling pathways. **(D)** Western blotting results showing that AFC significantly affected the expression of the GPX4-GSS/GSR-GGT axis, autophagy-associated proteins, and ferroptosis regulatory proteins in NSCLC cell lines.

Considering the importance of GPX4 in the regulation of ferroptosis, it is imperative to study regulatory networks downstream of the GPX4 gene. Using the protein interaction prediction tool STING VERSION11.0 (https://string-db.org/cgi/input.pl, ©STRING Consortium 2020), we analysed signalling networks downstream of GPX4. The results suggested that GPX4 was regulated by GSTO2 and regulated downstream GSS/GSR and GGT family proteins (Figure 5A). GO analysis showed that the expression levels of GPX4 significantly affected biological processes, including small molecule metabolic processes, glutathione metabolic processes, cellular biosynthetic processes, and cellular responses to chemical stimuli (Figure 5B). The expression levels of GPX4 significantly affected molecular functions, such as catalytic, hypoglycin A gamma-glutamyl transpeptidase, glutathione hydrolase, and antioxidant activities (Figure 5B). KEGG pathway analysis showed that GPX4 was highly correlated with the regulation of glutathione metabolism and the ferroptosis signalling pathway (Figure 5C). Eventually, the STING prediction results were validated by a western blot. The results suggested that the expression of cell proliferation-associated proteins (Ki67, CDK2, and CCND), autophagy-associated proteins (ATG3 and LC3A/B), and negative regulators of ferroptosis (GPX4 and FTH1) decreased significantly in AFC-treatedA549 and HCC827 cell lines after 24 h compared with the control group. The expression of GPX4 downstream was also significantly lower in AFC-treated lung cancer cell lines than in control cells (Figure 5D). The results indicated that AFC significantly affected the expression of the GPX4-GSS/GSR-GGT axis in NSCLC cell lines.

## Discussion

The morbidity and mortality associated with NSCLC are increasing each year, placing new demands on existing therapies and drugs 1-4. AFC is used as a food additive for iron supplementation 5-8. However, we discovered that AFC contains Fe^3+^. Fe^3+^ is convertible to Fe^2+^ *in vivo* and may induce ferroptosis 8. Therefore, the above results provide indications regarding whether a specific concentration of AFC can inhibit the proliferation of NSCLC. Through a cell proliferation inhibition test, a transwell assay, and flow cytometry analysis of the cell cycle and apoptosis, we confirmed that AFC at a specific concentration effectively inhibited the proliferation and invasion of lung cancer cell lines *in vitro*. This effect was achieved by appreciably inhibiting cell cycle progression and inducing cell death. In addition, we observed that AFC significantly induced the production of ROS and MDA in lung cancer cell lines while significantly reducing SOD and mitochondrial function. Therefore, AFC promotes oxidative stress in lung cancer cells. According to existing studies, oxidative stress provides the necessary conditions for ferroptosis and is a prerequisite for it 20-25. Subsequently, we systematically analysed the expression levels of ferroptosis-associated regulatory genes, autophagy initiation factors, and cell proliferation and death-associated factors. The results demonstrated that AFC significantly reduced the expression levels of cell growth factors, negative regulators of ferroptosis, and autophagy-associated factors. Therefore, the decrease in the proliferation capacity and the ability of cells to break down (autophagy) harmful substances and damaged subcellular organelles led to a specific type of cell death, namely ferroptosis. Once we demonstrated the mechanism of action of AFC, we further investigated the role of GPX4, a vital negative regulator of ferroptosis, in the overall process. A protein-protein interaction analysis revealed that GPX4 played a biological role through the regulation of the GSS/GSR complex and downstream GGT family proteins. When the expression of GPX4 changes, its biological activities, such as glutathione metabolic processes, cellular biosynthetic processes, cellular responses to chemical stimuli, and antioxidant activities, change accordingly, affecting the survival quality and physiological and biochemical activities of cells. These results partly explain the mechanism of AFC-induced ferroptosis in NSCLC cell lines.

Previous studies indicate that autophagy and ferroptosis are two different physiological phenomena in cells. Autophagy is the degradation of endogenous substrates through lysosomes, including damaged and senescent organelles, and the breakdown of proteins, amino acids, and fatty acids to meet the needs of cell energy metabolism and survival. The rate of autophagy is low in normal cells but increases when the extracellular environment changes. A large number of autophagosomes are produced, and they eventually induce cell death 28-30. Autophagy is abnormally high in tumour cells, which is assumed to be closely related to the abnormally active cell division and metabolism 28-31. However, many recent studies have demonstrated that autophagy and ferroptosis are not two independent events but are inextricably linked 31-35. Autophagy and ferroptosis are mutually reinforcing and interdependent, and they exhibit different interrelationships in different individuals, cells, and disease processes 33, 34. An et al. reported a positive correlation between ferroptosis and autophagy after autophagy-promoting rapamycin was loaded onto MnO_2_@HMCu_2_-xS nanocomposites and transfected into human breast cancer cells. It was speculated that autophagy is an indispensable physiological phenomenon in the process of ferroptosis in cells 36.Wei et al. reported that arsenic could damage mitochondria and induce pancreatic dysfunction and ferroptosis via mitochondrial ROS and autophagy-lysosomal disorder 37. However, Zhao also found that 15-lipoxygenase-1-PE-binding protein-1-generated ferroptotic phospholipids and 15-proferroptotic hydroperoxy-arachidonoyl-phosphatidylethanolamines promoted LC3-I lipidation to stimulate autophagy 38. This concurrent activation of autophagy protects cells from ferroptotic death and the release of mitochondrial DNA. Therefore, when there is an excess of iron ions, the autophagy rate decreases, leading to ferroptotic death 38. In addition, Liu et al. provided novel evidence that the interplay between the signals of the mechanistic target of rapamycin kinase and GPX4 modulates autophagy-dependent ferroptosis in human pancreatic cancer cells, and the down-regulation for GPX4 enhances the anti-cancer activity of rapamycin *in vitro* or *in vivo* by promoting ferroptosis and suppressing autophagy 39. Therefore, autophagy and ferroptosis are closely related. The relationship between them is not simply positive or negative, rather, different regulatory mechanisms are involved depending on the situation. The present study suggests that AFC significantly inhibits autophagy in NSCLC cell lines and promotes ferroptosis, which is not in accordance with the findings of other studies. We speculated that ferroptosis was induced by AFC because AFC first decreased the autophagy activity of lung cancer cells, resulting in their inability to digest and break down oxidative stress-damaged subcellular organelles or toxic substances. Therefore, the findings of this study further elucidate the relationship between ferroptosis and autophagy and provide new insights into the interaction between the two.

**Figure 6:**
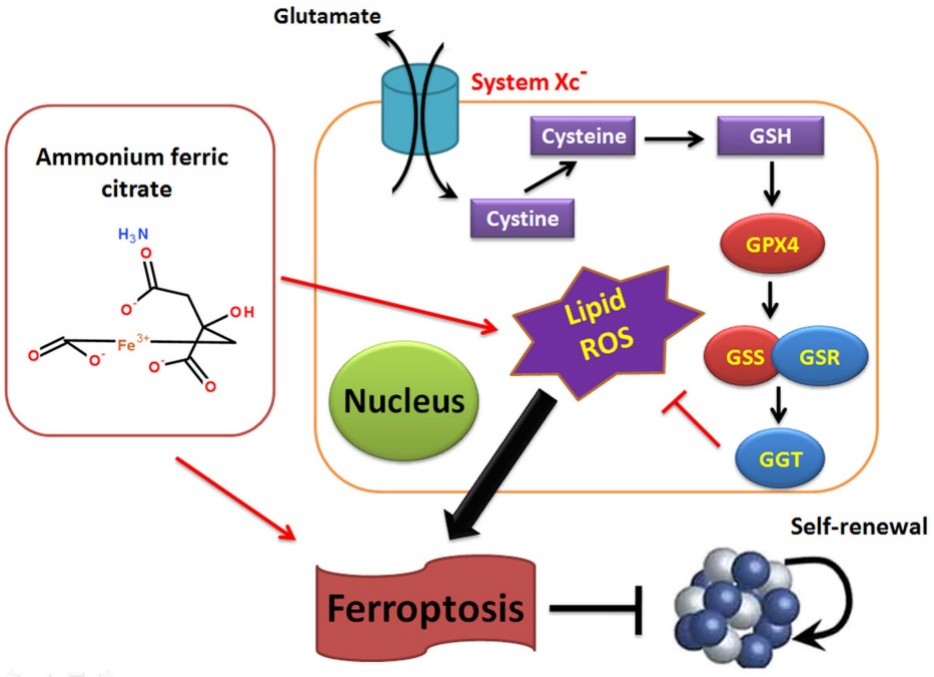
Ammonium ferric citrate induced ferroptosis in non-small-cell lung carcinoma through the inhibition of gpx4-gss/gsr-ggt axis activity.

Overall, this study verifies that AFC has the biological role of activating oxidative stress injury in NSCLC cell lines, leading to a decrease in autophagy and inducing ferroptosis. It also confirms that the GPX4-GSS/GSR-GGT axis is a crucial target of AFC-induced ferroptosis (Figure 6).

## Supplementary Material

Supplementary figures and table.
