# Supplementary material for: Ammonium Ferric Citrate induced Ferroptosis in Non-Small-Cell Lung Carcinoma through the inhibition of GPX4-GSS/GSR-GGT axis activity
Source: Int J Med Sci. 2021 Mar 3;18(8):1899–909. doi: 10.7150/ijms.54860 (PMC7976582; doi:10.7150/ijms.54860)
Supplement: Supplementary file 1 — Supplementary figures and table. [file ijmsv18p1899s1.pdf]

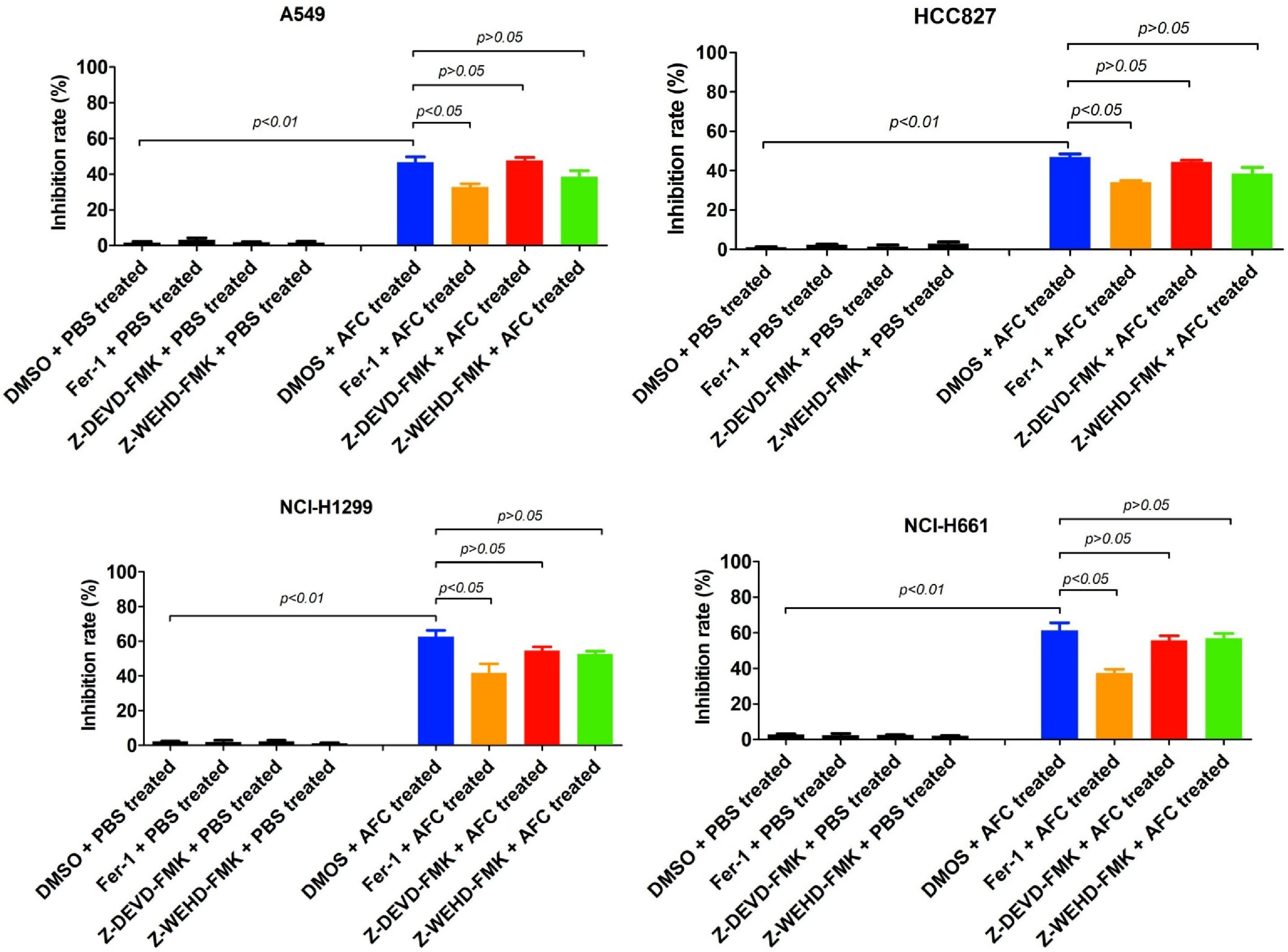
A549
HCC827
NCI-H1299
NCI-H661
Inhibition rate (%)
0
20
40
60
80
100
p<0.01
p<0.05
p>0.05
p>0.05
DMSO + PBS treated
Fer-1 + PBS treated
Z-DEVD-FMK + PBS treated
Z-WEHD-FMK + PBS treated
DMOS + AFC treated
Fer-1 + AFC treated
Z-DEVD-FMK + AFC treated
Z-WEHD-FMK + AFC treated

Figure S1. Rescue effect of different inhibitors on AFC induced cell proliferation inhibition in NSCLC cell lines

Z-DEVD-FMK is a specific caspase-3 inhibitor. Z-WEHD-FMK is a specific caspase-1/5 inhibitor. Ferrostatin-1 (Fer-1) is a specific ferroptosis inhibitor.

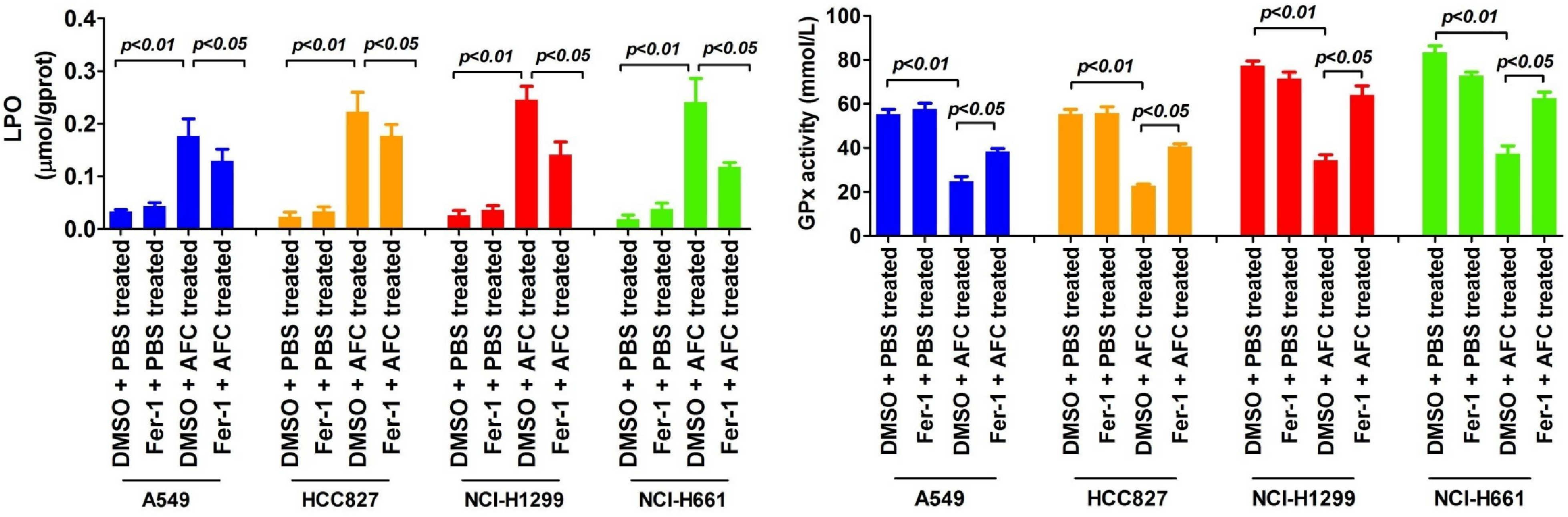

Figure S2. Ferrostatin 1 (Fer-1) reversed lipid peroxidation deposition in NSCLC cell lines

Table S1 qPCR Results

Cell line	GENE	PBS treated ($2^{-\Delta\Delta Ct}$)			AFC treated ($2^{-\Delta\Delta Ct}$)		
		1#	2#	3#	1#	2#	3#
A549	Endog	0.909	0.659	1.931	13.236	18.500	5.846
A549	Hsp1	0.474	0.533	0.650	4.112	3.778	2.763
A549	Rpl8	1.010	1.583	0.994	7.593	6.918	5.145
A549	Ppif	0.353	0.431	0.447	2.004	2.809	0.882
A549	Gclm	0.618	2.300	0.256	1.925	0.273	8.376
A549	Fsp1	0.714	0.847	0.927	2.505	2.093	1.850
A549	Lreb2	0.799	0.679	1.448	2.596	3.116	1.335
A549	Ftl	0.776	1.216	0.764	2.115	1.233	2.240
A549	Gclc	1.104	2.061	0.912	2.986	1.861	2.959
A549	Gss	1.781	3.086	1.585	3.136	1.494	4.064
A549	Becn1	0.912	1.468	0.873	1.639	1.293	1.283

A549	Chac1	0.886	1.187	1.020	1.530	1.253	1.154
A549	Fth1	0.880	1.641	0.728	1.440	0.819	1.564
A549	Atg12	1.133	1.589	1.246	1.729	1.523	1.212
A549	Hspb5	1.026	1.062	1.530	1.557	0.998	1.499
A549	Cs	0.824	1.344	0.779	1.109	0.649	1.170
A549	Nrf2	0.706	0.923	0.832	0.798	0.351	1.119
A549	Pgam5	2.276	3.121	2.559	2.811	2.007	2.431
A549	Bid	1.480	2.432	1.390	1.794	1.004	1.980
A549	Sat1	0.727	0.859	0.949	0.867	0.630	0.737
A549	Bak1	0.768	0.982	0.926	0.883	0.635	0.758
A549	Map1lc3b	0.904	1.940	0.650	0.973	0.361	1.621
A549	Tp53	1.081	1.239	1.455	1.213	1.065	0.853
A549	Ptgs2	0.749	0.900	0.962	0.763	0.409	0.877
A549	Cbs	0.844	1.226	0.896	0.807	0.438	0.918

A549	Cdk4	0.814	1.020	1.003	0.798	0.598	0.658
A549	Tfrc	0.992	1.250	1.215	0.940	0.833	0.656
A549	Cisd1	0.789	0.881	1.090	0.740	0.589	0.573
A549	Aifm1	2.270	3.460	2.297	1.924	0.937	2.440
A549	Bcl2	0.714	0.962	0.818	0.535	0.701	0.252
A549	Map1lc3c	1.516	2.607	1.359	1.155	0.527	1.562
A549	Ccnd	0.831	0.925	1.152	0.556	0.340	0.562
A549	Atp5g3	0.805	0.845	1.182	0.485	0.268	0.543
A549	Nfe2l2	1.227	1.385	1.678	0.739	0.758	0.445
A549	Ccne	1.082	0.933	1.937	0.650	0.509	0.513
A549	Cdk2	0.983	1.196	1.247	0.598	0.464	0.222
A549	Emc2	1.801	2.500	2.000	0.524	0.285	0.595
A549	Atg5	2.432	3.613	2.526	0.321	0.042	1.516
A549	Gls2	1.780	3.402	1.436	0.397	0.202	0.481

A549	Acsl4	1.925	2.211	2.584	0.361	0.247	0.327
A549	Htg4	2.598	3.571	2.914	0.348	0.445	0.168
A549	Ftc35	1.945	3.445	3.230	0.349	0.151	0.251
A549	Alox5	1.118	1.515	1.272	0.131	0.115	0.092
A549	Slc7a11	1.822	2.544	2.012	0.118	0.046	0.185
HCC827	Pgam5	0.367	0.395	0.380	17.238	20.902	18.575
HCC827	Htg4	0.321	0.346	0.333	4.182	11.020	6.643
HCC827	Gss	2.491	0.095	0.486	4.146	35.580	11.886
HCC827	Endog	0.345	2.633	0.952	41.363	5.550	14.827
HCC827	Rpl8	1.188	0.619	0.856	6.757	20.724	11.580
HCC827	Aifm1	0.368	0.396	0.381	11.378	0.712	2.785
HCC827	Atg5	2.550	0.050	0.356	0.669	17.633	3.361
HCC827	Acsl4	2.620	0.077	0.449	0.435	8.057	1.832
HCC827	Tp53	0.765	0.838	0.800	1.243	3.913	2.158

HCC827	Emc2	2.171	0.107	0.480	0.331	6.151	1.395
HCC827	Fth1	1.834	0.528	0.983	0.984	5.306	2.237
HCC827	Hspb5	1.220	0.583	0.843	0.774	3.722	1.661
HCC827	Gls2	0.613	0.386	0.486	1.267	0.818	0.996
HCC827	Nfe2l2	2.149	0.232	0.704	0.497	3.928	1.367
HCC827	Atg12	0.737	0.793	0.763	2.290	0.712	1.250
HCC827	Map1lc3c	0.551	0.593	0.570	1.465	0.712	1.000
HCC827	Lreb2	1.202	0.978	1.082	1.565	1.950	1.710
HCC827	Becn1	1.100	0.820	0.948	0.872	2.248	1.370
HCC827	Ftl	0.975	1.276	1.114	1.679	1.385	1.492
HCC827	Fsp1	1.171	1.258	1.212	1.654	1.056	1.294
HCC827	Bid	0.564	0.607	0.584	0.553	0.712	0.614
HCC827	Gclc	2.313	0.266	0.783	1.775	0.712	1.100
HCC827	Cbs	1.159	0.908	1.024	0.508	1.669	0.901

HCC827	Slc7a11	2.157	0.105	0.475	0.199	1.850	0.594
HCC827	Chac1	0.942	1.014	0.976	0.644	1.257	0.881
HCC827	Ccne	0.544	1.177	0.799	0.736	0.806	0.754
HCC827	Gclm	1.103	1.783	1.400	1.310	1.135	1.193
HCC827	Cs	0.836	1.322	1.049	1.081	0.721	0.864
HCC827	Tfrc	0.841	0.905	0.871	0.553	0.712	0.614
HCC827	Ftc35	2.041	0.153	0.148	0.303	0.712	0.614
HCC827	Map1lc3b	0.844	1.087	0.956	0.772	0.533	0.628
HCC827	Ccnd	1.102	0.984	1.040	0.642	0.629	0.622
HCC827	Cdk4	0.983	1.151	1.062	0.575	0.608	0.579
HCC827	Bak1	1.090	1.167	1.126	0.709	0.542	0.607
HCC827	Cisd1	1.097	1.098	1.096	0.545	0.600	0.560
HCC827	Alox5	0.729	0.823	0.773	0.280	0.467	0.354
HCC827	Hsp1	1.925	1.734	1.824	0.852	0.758	0.787

HCC827	Cdk2	0.854	0.908	0.879	0.357	0.382	0.361
HCC827	Sat1	1.369	1.037	1.189	0.280	0.763	0.452
HCC827	Nrf2	1.274	1.182	1.226	0.454	0.500	0.466
HCC827	Atp5g3	0.816	1.419	1.074	0.510	0.245	0.346
HCC827	Bcl2	1.331	1.104	1.210	0.250	0.508	0.349
HCC827	Ptgs2	0.694	1.924	1.154	0.493	0.194	0.302
HCC827	Ppif	2.720	2.210	2.448	0.149	0.460	0.256
